# Microsatellite genotyping and computational inference for genetic management of the Eurasian wild boar (*Sus scrofa* L., 1758): strengths, limitations, and integrative applications

**DOI:** 10.5114/bta/226392

**Published:** 2026-09-09

**Authors:** Wojciech Lipa, Laura Iacolina, Artur Dzialuk

**Affiliations:** 1 Department of Microbiology and Immunobiology, Faculty of Biological Sciences, Kazimierz Wielki University, Bydgoszcz, Poland; 2 Doctoral School, Kazimierz Wielki University, Bydgoszcz, Poland; 3 Department of Veterinary Medicine, University of Sassari, Sassari, Italy; 4 National Biodiversity Future Center – NBFC, Palermo, Italy; 5 Department of Chemistry and Bioscience, Aalborg University, Aalborg, Denmark; 6 Department of Genetics, Faculty of Biological Sciences, Kazimierz Wielki University, Bydgoszcz, Poland

**Keywords:** Eurasian wild boar, population structure, microsatellites, admixture, connectivity, computational inference

## Abstract

Microsatellite markers, commonly referred to as short tandem repeats (STRs) or simple sequence repeats, have been extensively utilized in population genetic studies of the Eurasian wild boar (*Sus scrofa* L., 1758), particularly for assessing genetic diversity, population structure, connectivity, relatedness, and admixture with domestic pigs (*Sus scrofa domesticus* L., 1758). Despite the increasing availability of single nucleotide polymorphism (SNP)-based and whole-genome approaches, STR datasets remain valuable because of their high polymorphism, relatively low cost, and compatibility with legacy monitoring data. This review provides a critical synthesis of the use of microsatellite genotyping in wild boar research, with particular emphasis on the computational inference frameworks used to translate multi-locus genotypes into management-relevant information. We discuss the application of STR data to diversity and inbreeding metrics, clustering and assignment analyses, effective population size and bottleneck inference, parentage and kinship reconstruction, admixture detection, landscape genetics, and disease-related interpretation. Special emphasis is placed on the assumptions and limitations of microsatellite-based inference, including marker panel size and comparability, sampling design, allele binning, null alleles, genotyping error, allele-size homoplasy, and the temporal scale of interpretation. The review highlights that STRs provide substantial insights into recent and fine-scale population processes, regional monitoring, and preliminary admixture screening, whereas dense SNP panels or whole-genome data are required for precise introgression quantification, adaptive inference, complex demographic reconstruction, and deep phylogeographic analysis. Used within an integrative framework that combines genomic, landscape, epidemiological, and long-term monitoring data, STRs remain a cost-effective and informative component of genetic monitoring for *S. scrofa* across heterogeneous and human-modified landscapes.

## Introduction

The Eurasian wild boar (*Sus scrofa* L., 1758) is a highly adaptable ungulate with a broad distribution, and its populations are increasingly influenced by several factors such as habitat fragmentation, hunting pressure, translocations, hybridisation with domestic pigs, and disease-related disturbances, including African swine fever (ASF) (Frantz et al. 2013; Goedbloed et al. 2013; Griciuvienė et al. 2022; Massei et al. 2015; Meletiadis et al. 2025; Scandura et al. 2011; Simon et al. 2024). These processes create management challenges that can be addressed only through reliable information on genetic diversity, population structure, dispersal, connectivity, and admixture (Griciuvienė et al. 2021; Lecis et al. 2022; Mihalik et al. 2020; Sawai et al. 2023). Consequently, genetic data are relevant not only for describing population differentiation but also for defining management-relevant units, identifying potential barriers or corridors for species movement, monitoring temporal change, and interpreting population processes that may impact disease spread and control strategies (Goicolea et al. 2024; Griciuvienė et al. 2022; Meletiadis et al. 2025; Pepin et al. 2021; Simon et al. 2024).

Microsatellite markers, also known as STRs or simple sequence repeats (SSRs), are pivotal components in the study of wild boar population genetics because they are highly polymorphic, co-dominant, relatively cost-effective to genotype, and compatible with various legacy datasets (Bruford and Wayne 1993; Guichoux et al. 2011; Hauser et al. 2021). In *S. scrofa*, STR panels have been used to estimate genetic diversity, infer population structure and connectivity, assess relatedness and parentage, and detect admixture between wild boar and domestic pigs (Anderson et al. 2020; Costa et al. 2012; Frantz et al. 2013; Podgórski et al. 2014; Sawai et al. 2023; Simon et al. 2024). Their continued value is particularly evident in regional monitoring programmes and in studies where comparability with previously generated datasets is important (Hauser et al. 2021; Osborne et al. 2023; Salih et al. 2025).

Although microsatellite-based inference offers valuable knowledge, its robustness is not universally applicable. The reliability of this approach is influenced by marker panel composition, sampling design, allele scoring, missing data, and model assumptions (Chapuis and Estoup 2007; Guichoux et al. 2011; Kierepka et al. 2016; Putman and Carbone 2014). In the present review, STRs are therefore treated primarily as markers for recent, management-relevant processes, whereas deeper evolutionary reconstruction, adaptive inference, and precise quantification of introgression are considered domains that generally require denser genomic or integrative approaches (De Jong et al. 2023; Frantz et al. 2013; Mary et al. 2022; Niedziałkowska et al. 2021; Scandura et al. 2008; Wang et al. 2025).

This review critically synthesises the use of microsatellite markers in studies on the Eurasian wild boar, prioritizing their contribution to genetic monitoring and management. Rather than treating STRs only as a marker system, we focus on the types of inference supported by them: estimation of diversity and inbreeding-related metrics, detection of population structure and connectivity, assignment and admixture analysis, parentage and kinship inference, bottleneck and effective population size estimation, and landscape-genetic interpretation. We also evaluate the conditions under which microsatellite-based conclusions are reliable, their limitations, and the requirement for complementary SNP-based or genome-wide approaches.

The review uses a narrative approach while adhering to an explicit literature selection strategy. We considered empirical studies using STR/SSR genotyping in *S. scrofa*, particularly those addressing population-level processes, management-relevant inference, hybridisation with domestic pigs, disease-related demographic change, or landscape connectivity. Relevant studies were identified from the Web of Science, Scopus, and Google Scholar databases using the following combination of key terms: ‘*Sus scrofa*’, ‘wild boar’, ‘microsatellite’, ‘STR’, ‘SSR’, ‘population structure’, ‘gene flow’, ‘admixture’, ‘hybridisation’, ‘kinship’, ‘parentage’, ‘effective population size’, ‘bottleneck’, ‘landscape genetics’, ‘African swine fever’, and ‘management’. Backward and forward citation tracking was employed to include key methodological and species-specific studies. Recent publications were prioritized, particularly from 2020 onward, while older studies that provided conceptual foundations, long-term comparability, or historically important datasets were retained.

## Microsatellite markers in wild boar genetics: biological basis and analytical constraints

Microsatellites are tandemly repeated DNA motifs characterised by allelic variations that predominantly occur from changes in repeat number (Ellegren 2004; Kruglyak et al. 1998; Levinson and Gutman 1987). This phenomenon of length polymorphism is primarily attributed to replication slippage, with unequal crossing-over and point mutations contributing less frequently to repeat variation and motif instability (Antão-Sousa et al. 2023; Ellegren 2004; Fan and Chu 2007; Kruglyak et al. 1998; Levinson and Gutman 1987). These mechanisms generate significant allelic diversity; however, they also explain the necessity for careful treatment of mutation models, homoplasy, and allele scoring during downstream analyses of STR data (Bruford and Wayne 1993; Costa et al. 2012; Frantz et al. 2013; Guichoux et al. 2011; Sawai et al. 2023). The same mutational properties that make microsatellites informative also impose limits on their interpretation (Guichoux et al. 2011; Putman and Carbone 2014; Selkoe and Toonen 2006). Stepwise-like mutation, allele-size homoplasy, null alleles, allele binning, and scoring error can affect estimates of diversity, population structure, relatedness, and admixture, particularly when small or heterogeneous STR panels are compared across broad spatial or temporal scales (Chapuis and Estoup, 2007; Estoup et al. 2002; Guichoux et al. 2011; Putman and Carbone 2014). Therefore, these constraints should not be considered as justifications for disregarding STRs; rather, they should be perceived as conditions that define the appropriate scale and confidence of microsatellite-based inference (Mihalik et al. 2020; Griciuvienė et al. 2022; Meletiadis et al. 2025). Consequently, microsatellite-based inference should be interpreted within the context of marker choice, sampling design, and assumptions of the utilized computational methods (Guichoux et al. 2011; Putman and Carbone 2014; Selkoe and Toonen 2006).

Microsatellites are frequently regarded as neutral markers in population genetic studies; however, STRs can also be located in transcribed, regulatory, and coding regions of the genome, potentially impacting gene regulation, genome organisation, or protein structure in a locus-specific manner (Bagshaw 2017; Reinar et al. 2024; Wright and Todd 2023). For management-associated studies of wild boar, however, the primary value of microsatellites is not their functional implications but rather their capacity to provide information-rich content for recent demographic and spatial processes (Griciuvienė et al. 2022; Hauser et al. 2021; Meletiadis et al. 2025; Sawai et al. 2023). This makes STRs useful for regional genetic monitoring, particularly in situations where maintaining continuity with established legacy datasets is important (Hauser et al. 2021; Osborne et al. 2023; Salih et al. 2025). Sequence-based microsatellite genotyping approaches, including SSRseq, can improve allele calling, reproducibility, and cross-study comparability by reducing artefacts associated with fragment-length scoring (De Barba et al. 2017; Lepais et al. 2020; Salih et al. 2025; Šarhanová et al. 2018). In this context, their main contribution is methodological standardisation and continuity with legacy STR datasets rather than a change in the biological scale of inference (Hauser et al. 2021; Meletiadis et al. 2025; Sawai et al. 2023; Simon et al. 2024). Figure 1 summarises the workflow linking sampling, microsatellite genotyping, quality control, computational inference, and management interpretation.

**Figure 1:**
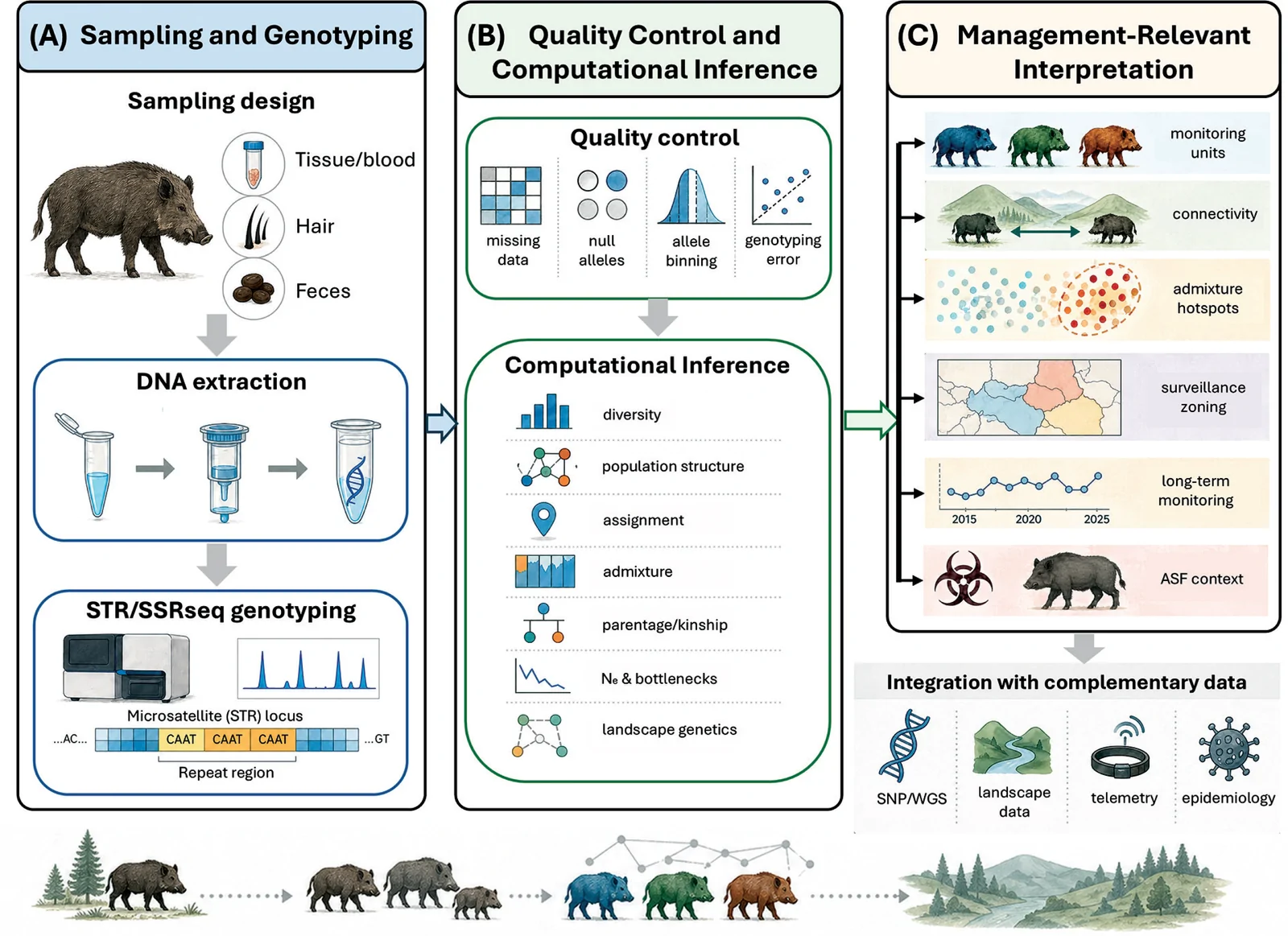
Conceptual workflow linking microsatellite genotyping to management-relevant inference in Eurasian wild boar. ASF – African swine fever, Ne – effective population size, SNP – single nucleotide polymorphism, SSR – simple sequence repeat, STR – short tandem repeat, WGS – whole-genome sequencing

## Microsatellite datasets in Eurasian wild boar studies: scope, inference goals, and management relevance

Microsatellite datasets have been widely used to address population-level questions in the Eurasian wild boar, particularly where management requires insights regarding genetic diversity, spatial structure, connectivity, dispersal, and admixture (Frantz et al. 2013; Sawai et al. 2023; Scandura et al. 2011; Simon et al. 2024). Across Europe and Asia, STR-based studies have contributed to the delineation of genetically differentiated populations, identification of barriers to gene flow, assessment of regional connectivity, and detection of wild boar × domestic pig admixture (Choi et al. 2014; Frantz et al. 2013; Lecis et al. 2022; Sawai et al. 2023; Simon et al. 2024). These applications are especially relevant in landscapes affected by hunting pressure, habitat fragmentation, translocations, and demographic changes due to diseases, including ASF (Goicolea et al. 2024; Griciuvienė et al. 2021, 2022; Meletiadis et al. 2025; Simon et al. 2024).

The main inferential goals of microsatellite-based studies in *S. scrofa* can be grouped into several recurring categories. First, STR panels have been used to estimate within-population diversity, including observed and expected heterozygosity, allelic richness, and inbreeding-related indices, providing indicators of demographic perturbation or restricted connectivity (Griciuvienė et al. 2022; Mihalik et al. 2020; Rodrigáñez et al. 2008). Second, allele-frequency differentiation and individual assignment approaches have been used to infer population structure, dispersal limitation, and management-relevant connectivity across heterogeneous landscapes (Lecis et al. 2022; Meletiadis et al. 2025; Mihalik et al. 2020; Sawai et al. 2023; Simon et al. 2024). Third, microsatellites, often in combination with other marker systems, have supported the detection of admixture and introgression between wild boar and domestic pigs, particularly in regions affected by releases, translocations, or contact with free-ranging domestic animals (Böheim et al. 2023; Frantz et al. 2013; Goedbloed et al. 2013; Johansson et al. 2026; Mary et al. 2022; Schleimer et al. 2022). Finally, STR-based parentage and kinship analyses have provided insights into relatedness, reproductive structure, and aspects of social organisation, although these applications require dense sampling and careful monitoring of genotyping errors (Podgórski et al. 2014; Poteaux et al. 2009; Waits and Paetkau 2005; Zemanova 2021).

From a management perspective, these applications are useful because they translate individual genotypes into population-level indicators (Figure 1). Genetic structure can help identify demographically connected or isolated units, connectivity analyses can indicate potential dispersal corridors or barriers, and admixture estimates can reveal areas where domestic ancestry may affect conservation or management objectives (Frantz et al. 2013; Lecis et al. 2022; Sawai et al. 2023; Simon et al. 2024). In systems affected by diseases, including ASF, although neutral genetic structure does not directly reconstruct pathogen transmission, it can provide complementary information on host connectivity and spatial population subdivision relevant to surveillance and control planning (Goicolea et al. 2024; Meletiadis et al. 2025; Pepin et al. 2021; Simon et al. 2024).

Collectively, published STR studies indicate that wild boar genetic structure is rarely explained by a single driver. Geography and isolation by distance often provide a broad background pattern, whereas fragmentation, insularity, roads, hunting-related translocations, and local demographic perturbations may increase differentiation at regional scales (Lecis et al. 2022; Mihalik et al. 2020; Sawai et al. 2023; Simon et al. 2024). Domestic pig ancestry is also spatially heterogeneous and may reflect recent hybridisation, older introgression, or incomplete separation between wild and domestic gene pools rather than a simple wild versus domestic contrast (Böheim et al. 2023; Frantz et al. 2013; Mary et al. 2022; Schleimer et al. 2022). These patterns indicate that STR datasets are most informative when interpreted comparatively across management contexts, particularly in relation to diversity, structure, admixture, and connectivity, rather than as isolated methodological outputs.

## Computational inference from microsatellite data in wild boar research

Microsatellite genotyping is relevant in management context only when multi-locus genotypes are translated into explicit population genetic inference (Guichoux et al. 2011; Putman and Carbone 2014). In research on wild boar, the principal outputs are estimates of diversity and inbreeding-related indices, population clusters and assignments, admixture probabilities, relatedness estimates, effective population size, bottleneck signals, and landscape–genetic associations (Anderson and Thompson 2002; Do et al. 2014; Jones and Wang 2010; Meletiadis et al. 2025; Mihalik et al. 2020; Pritchard et al. 2000; Sawai et al. 2023; Simon et al. 2024). The same dataset may support some of these goals better than others; therefore, this section summarises how analytical choices affect inference rather than repeating a general catalogue of methods (Meirmans 2015; Putman and Carbone 2014). Quality control and marker panel comparability are the two main prerequisites. Allele-calling errors, stutter, large-allele dropout, null alleles, missing data, and inconsistent allele binning can introduce bias in heterozygosity determination, Hardy-Weinberg tests, and estimation of structure, relatedness, and admixture (Figure 1) (Chapuis and Estoup 2007; Dakin and Avise 2004; Guichoux et al. 2011; Putman and Carbone 2014). Hence, checks for missing data, deviations from Hardy-Weinberg equilibrium, linkage disequilibrium, scoring consistency, and null alleles should be reported, and temporal or cross-study comparisons should be restricted to comparable marker sets and sampling schemes (Bonin et al. 2004; Kierepka et al. 2016; Waits and Paetkau 2005). Sequence-based genotyping can reduce fragment-scoring artefacts; however, it should be viewed as a standardisation tool rather than a substitute for explicit validation (De Barba et al. 2017; Lepais et al. 2020; Salih et al. 2025). Observed and expected heterozygosity, allelic richness, private alleles, and inbreeding coefficient (F_IS_) are useful for comparing populations, identifying local loss of variation, and detecting possible demographic perturbation (Griciuvienė et al. 2021, 2022; Mihalik et al. 2020; Rodrigáñez et al. 2008). In wild boar, however, heterozygote deficiency may result not only from inbreeding but also from Wahlund effects, social group pooling, null alleles, or inconsistent spatial sampling; consequently, interpretation is more robust when diversity indices are evaluated together with spatial structure and marker diagnostics (Chapuis and Estoup 2007; Hale et al. 2012; Podgórski et al. 2014; Putman and Carbone 2014). Bottleneck and effective population size estimates can indicate recent demographic disturbances caused by factors such as diseases, hunting, or isolation; however, weak or historical changes may be overlooked, particularly when utilizing small marker panels, low sample sizes, or in the presence of immigration (Cornuet and Luikart 1996; Do et al. 2014; Garza and Williamson 2001; Peery et al. 2012; Waples and Do 2010).

Population structure and assignment analyses are most effective when they correlate allele frequency variations with specific spatial hypotheses. Bayesian clustering, multivariate approaches, and assignment tests can identify differentiated groups, likely migrants, and areas of exchange (Evanno et al. 2005; Jombart et al. 2010; Manel et al. 2005; Pritchard et al. 2000; Sawai et al. 2023; Simon et al. 2024). In *S. scrofa*, these outputs should be interpreted as probabilistic evidence of connectivity or differentiation rather than fixed population boundaries, because isolation by distance, inconsistent sampling, family structure, translocations, and recent mortality can generate similar clustering patterns (Frantz et al. 2009; Meirmans 2012; Puechmaille 2016; Putman and Carbone 2014). Assignment accuracy also declines when reference populations are missing or are weakly differentiated (Cornuet and Luikart 1996; Paetkau et al. 2004; Rannala and Mountain 1997). Admixture analyses play a critical role in identifying wild boar × domestic pig gene flow. STR panels are effective tools for screening recent hybrids or populations with clear domestic ancestry, provided that adequate wild and domestic reference samples are available (Anderson et al. 2020, 2021; Anderson and Thompson 2002; Frantz et al. 2013; Vähä and Primmer 2006). However, the efficacy of these panels declines for low-level, old, or complex introgression; consequently, microsatellite estimates should be treated as screening evidence and interpreted cautiously in regions where historical introgression, recent releases, and incomplete differentiation may coexist (Böheim et al. 2023; Goedbloed et al. 2013; Mary et al. 2022; Schleimer et al. 2022). Parent-age, kinship, and relatedness analyses exploit the high individual-level discriminatory power of STRs and have been used to study reproductive skew, kin-structured groups, sex-biased dispersal, and local social organisation (Blouin 2003; Costa et al. 2012; Podgórski et al. 2014; Poteaux et al. 2009). These analyses have significance in identifying family-level structure that can influence dispersal, recruitment, or contact rates; however, complete pedigree reconstruction is rarely realistic in open wild boar populations with unsampled relatives (Jones and Wang 2010; Marshall et al. 1998; Waits and Paetkau 2005; Wang 2011). Landscape–genetic analyses connect genetic differentiation with geographic distance, habitat configuration, and anthropogenic barriers (Holderegger and Wagner 2008; Manel et al. 2003; Storfer et al. 2006). Such analyses in wild boar are particularly relevant in fragmented or disease-affected landscapes, where roads, forests, agricultural matrices, hunting regimes, and control barriers may influence movement and gene flow (Lecis et al. 2022; Sawai et al. 2023; Simon et al. 2024). Hence, in the context of ASF, STR data should be interpreted as host-connectivity evidence rather than direct evidence of pathogen transmission, with epidemiological conclusions requiring integration with pathogen, movement, and surveillance data (Goicolea et al. 2024; Mazloum et al. 2023; Meletiadis et al. 2025; Pepin et al. 2021). The main inference targets, analytical outputs, assumptions, and management implications of STR-based analyses are summarised in Table 1.

**Table 1:** Main inference targets, analytical frameworks, and limitations of microsatellite-based studies in Eurasian wild boar

Inference target	Common outputs/approaches	Key assumptions and requirements	Main limitations	Management relevance	Selected references
Genetic diversity	H_o_, H_e_, allelic richness, private alleles; population-level summary statistics	Representative sampling, sufficient sample size, polymorphic and consistently scored loci	Sensitive to sample size, null alleles, missing data, and differences among marker panels	Detection of reduced diversity, isolation, or demographic perturbation	Rodrigáñez et al. 2008; Costa et al. 2012; Guichoux et al. 2011; Putman and Carbone 2014; Griciuvienė et al. 2022
Inbreeding-related patterns	F_IS_, heterozygote deficiency, relatedness-based indicators	Correct population definition, low genotyping error, limited Wahlund effects	Heterozygote deficiency may reflect null alleles, substructure, or sampling artefacts rather than biological inbreeding	Identification of populations requiring closer genetic monitoring	Guichoux et al. 2011; Putman and Carbone 2014
Population structure	F_ST_, Bayesian clustering, DAPC, assignment tests	Spatially explicit sampling, informative loci, biologically meaningful grouping of samples	Clusters may reflect isolation by distance, family structure, inconsistent sampling, or hierarchical structure	Delineation of management-relevant units and differentiated population segments	Pritchard et al. 2000; Evanno et al. 2005; Jombart et al. 2010; Mihalik et al. 2020; Simon et al. 2024
Connectivity and dispersal	Assignment tests, migrant detection, isolation-by-distance analyses, landscape connectivity patterns	Well-sampled reference populations, suitable spatial scale, sufficient differentiation among areas	Genetic connectivity reflects multi-generational movement and may not capture recent dispersal; unsampled source populations reduce accuracy	Identification of corridors, barriers, and recolonisation routes and determination of the appropriate spatial scale of control	Poteaux et al. 2009; Sawai et al. 2023; Simon et al. 2024
Admixture and hybridisation	Admixture proportions, hybrid-class assignment, detection of domestic ancestry	Appropriate wild and domestic reference samples, informative loci, adequate number of markers	Limited power for weak, old, or complex introgression compared to SNP/WGS data	Identification of individuals’ legal status and of introgression hotspots	Anderson and Thompson 2002; Frantz et al. 2013; Goedbloed et al. 2013; Mary et al. 2022; Schleimer et al. 2022; Böheim et al. 2023
Parentage and kinship	Parentage assignment, kinship reconstruction, relatedness coefficients	High marker polymorphism, low genotyping error, dense sampling of candidate relatives	Incomplete sampling and genotyping error can bias pedigree or relatedness reconstruction	Interpretation of social structure, dispersal, reproductive patterns, and local recruitment	Marshall et al. 1998; Jones and Wang 2010; Wang 2011; Podgórski et al. 2014
Effective population size and bottlenecks	Contemporary N_e_, heterozygosity-excess tests, allele frequency mode shift, M-ratio	Adequate sample size, sufficient loci, model assumptions approximately met	Often limited statistical power with small STR panels; demographic signals may be masked by migration or sampling effects	Assessment of demographic perturbation caused by disease, hunting, isolation, or population control	Cornuet and Luikart 1996; Garza and Williamson 2001; Do et al. 2014
Landscape-genetic inference	Association between genetic differentiation and landscape resistance, barrier, or corridor modelling	Spatially balanced sampling, relevant environmental layers, explicit spatial modelling	Correlation does not imply causation; genetic structure may reflect historical rather than current connectivity	Landscape-level planning, corridor assessment, and identification of barriers to gene flow	Lecis et al. 2022; Sawai et al. 2023; Simon et al. 2024
Disease-related interpretation	Host population structure and connectivity interpreted in epidemiological context	Integration with epidemiological, spatial, ecological, or pathogengenomic data	STRs do not reconstruct pathogen transmission chains or short-term outbreak dynamics	Support for surveillance zoning, host-connectivity interpretation, and long-term monitoring in ASF-affected systems	Pepin et al. 2021; Griciuvienė et al. 2022; Meletiadis et al. 2025

ASF – African swine fever, DAPC – discriminant analysis of principal components, FIS – inbreeding coefficient, FST – fixation index, He – expected heterozygosity, Ho – observed heterozygosity, Ne – effective population size, SNP – single nucleotide polymorphism, STR – short tandem repeat, WGS – whole-genome sequencing.

## Management interpretation of STR-based inference in Eurasian wild boar

At an applied interpretative level, STR-based studies reveal that the genetic structure of wild boar populations is influenced by a complex interplay of multiple factors, including natural dispersal, landscape permeability, demographic disturbance, and human-mediated movement, rather than by a single recurrent mechanism (Lecis et al. 2022; Sawai et al. 2023; Scandura et al. 2011; Simon et al. 2024). The management value of these studies is therefore mainly comparative: microsatellite data can indicate whether (1) populations are broadly connected, (2) local diversity is reduced, (3) domestic ancestry is concentrated in particular areas, and (4) the observed structure corresponds to management zones or disease-control boundaries (Böheim et al. 2023; Griciuvienė et al. 2022; Lecis et al. 2022; Simon et al. 2024).

The interpretation of diversity, structure, and admixture must remain context-dependent. Reduced heterozygosity or allelic richness may not only indicate isolation, demographic disturbance, or population control, but it may also reflect sampling scale, Wahlund effects, null alleles, or genotyping artefacts (Chapuis and Estoup 2007; Griciuvienė et al. 2022; Hale et al. 2012; Putman and Carbone 2014). Similarly, genetic clusters may represent differentiated management units in fragmented, insular, or disease-affected landscapes; however, they may also reflect connectivity gradients or inconsistent sampling in continuous populations (Frantz et al. 2009; Goicolea et al. 2024; Meirmans 2012; Puechmaille 2016). Admixture with domestic pigs is likewise informative for identifying admixed individuals and domestic ancestry hotspots, although STRs are better suited for screening and geographic targeting than for resolving ancestry tracts or functional consequences, which require genome-wide evidence (Böheim et al. 2023; Mary et al. 2022; Schleimer et al. 2022). An individual-level perspective is provided through kinship-based analyses that show how social organisation, female kin associations, and sex-biased dispersal can structure genetic variation at fine spatial scales (Gamelon et al. 2025; Podgórski et al. 2014; Poteaux et al. 2009). This is relevant for disease ecology because kin structure may influence local contact opportunities, although genetic relatedness should be interpreted as one layer of host ecology rather than as direct evidence of pathogen transmission (Pepin et al. 2021). Therefore, the main unresolved issues are applied and comparative: how rapidly wild-boar populations regain diversity after ASF-related decline, whether apparent barriers reflect current restrictions or historical structure, how domestic introgression affects management-relevant traits, and how well genetic connectivity corresponds to movement over epidemiological timescales. Addressing these issues will require repeated sampling, harmonised marker panels or bridge datasets, and integration of STR information with SNP/whole-genome sequencing (WGS), telemetry, landscape resistance models, and pathogen data (Goicolea et al. 2024; Hauser et al. 2021; Meletiadis et al. 2025; Osborne et al. 2023; Salih et al. 2025).

## Phylogeography and evolutionary relationships

Microsatellite markers have contributed to studies on phylogeographic and evolutionary relationships in *S. scrofa* by revealing recent or intermediate-scale spatial differentiation, secondary contact, and population subdivision (Alexandri et al. 2017; Kusza et al. 2014; Scandura et al. 2008, 2011; Veličković et al. 2016). The significance of these markers mainly depends on the context: STRs can show how contemporary structure aligns with broader biogeographic patterns, especially when combined with geographic, ecological, and historical data (Lecis et al. 2022; Scandura et al. 2008, 2011; Veličković et al. 2016). Because high mutation rates, allele-size homoplasy, and stepwise-like mutation can obscure older demographic signals, microsatellitebased phylogeographic interpretations should be utilized as evidence for recent structuring rather than as stand-alone reconstructions of ancient divergence, glacial refugia, or long-term lineage history (de Jong et al. 2023; Estoup et al. 2002; Putman and Carbone 2014; Schlotterer 2000).

Integrative European studies using mitochondrial, Y-chromosome, SNP, and whole-genome data have refined our understanding of major lineages, contact zones, and Pleistocene-Holocene demographic processes (De Jong et al. 2023; Niedziałkowska et al. 2021; Scandura et al. 2011). STR datasets complement this evidence by resolving recent local structure and admixture among neighbouring populations, particularly where historical processes interact with habitat fragmentation, translocations, hunting management, and contact with domestic pigs (Frantz et al. 2013; Goedbloed et al. 2013; Mary et al. 2022; Schleimer et al. 2022). These findings indicate that the relationship between wild boars and domestic pigs should not be interpreted as a simple binary distinction. Domestic ancestry may reflect recent hybridisation, historical introgression, or incomplete differentiation between wild and domestic gene pools and therefore should be evaluated at both regional and genomic scales (Böheim et al. 2023; Mary et al. 2022; Schleimer et al. 2022).

## Strengths, limitations, and future directions

In research on Eurasian wild boar, the relevance of microsatellites can be attributed to a combination of several characteristics, including high polymorphism, low cost, technical accessibility, and compatibility with historical datasets (Guichoux et al. 2011; Hauser et al. 2021; Osborne et al. 2023; Salih et al. 2025). These features make STRs particularly useful for regional monitoring, temporal comparisons based on shared panels, preliminary admixture screening, and individual-level analyses such as kinship or parentage (Böheim et al. 2023; Costa et al. 2012; Griciuvienė et al. 2022; Putman and Carbone 2014; Simon et al. 2024). However, their main limitations are associated with scale and comparability: conclusions are more robust when marker panels, sampling design, allele scoring, and population grouping are sufficiently transparent to allow meaningful comparison across regions and time (Bonin et al. 2004; Guichoux et al. 2011; Selkoe and Toonen 2006; Van Oosterhout et al. 2004).

Instead of merely comparing STR with SNP/WGS approaches, future studies should apply these marker systems at the complementary levels of resolution according to the biological question, management context, and the required inferential scale. Legacy or lowcost STR panels can ensure ongoing monitoring and identify populations, contact zones, or management situations that require higher-resolution genomic analysis, whereas SNP/WGS approaches are better suited to issues related to adaptive variation, complex demographic history, or fine-scale introgression (De Jong et al. 2023; Goedbloed et al. 2013; Mary et al. 2022; Schleimer et al. 2022). Figure 2 summarises this decision framework. In fragmented or ASF-affected landscapes, the applied value of STR-based connectivity will depend on integration with ecological, movement, hunting, carcass, pathogen, and spatiotemporal data, so that genetic monitoring contributes to management inference rather than remaining a descriptive endpoint (Alkhamis et al. 2018; Goicolea et al. 2024; Meletiadis et al. 2025; Pepin et al. 2021; Simon et al. 2024). This integrative direction is the most likely route for converting STR-based monitoring from descriptive population genetics into decision-relevant management evidence.

**Figure 2:**
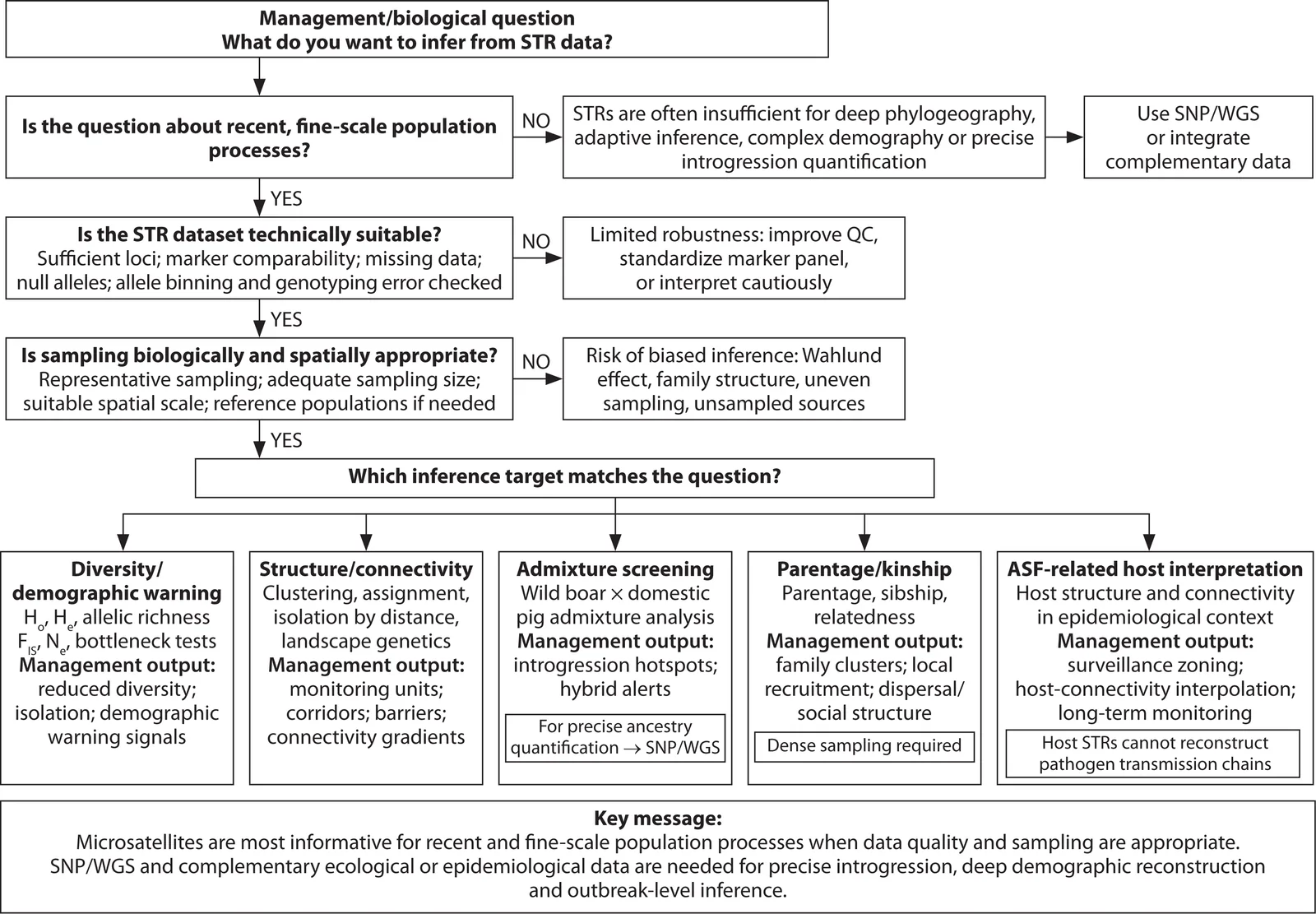
Decision framework for matching microsatellite data to management-oriented inference in Eurasian wild boar. ASF – African swine fever, FIS – inbreeding coefficient, H_e_ – expected heterozygosity, H_o_ – observed heterozygosity, N_e_ – effective population size, QC – quality control, SNP – single nucleotide polymorphism, STR – short tandem repeat, WGS – whole-genome sequencing

## Conclusions

Overall, microsatellite markers remain useful for genetic management of the Eurasian wild boar when their application corresponds to recent, managementrelevant questions and to comparable datasets. These markers are highly relevant in supporting regional-scale genetic monitoring, broad assessment of diversity and population structure, preliminary screening for wild boar × domestic pig admixture, and kinship-oriented analyses, particularly where continuity with legacy STR datasets is critical.

Multiple studies have shown that wild boar genetic diversity, connectivity, and admixture reflect interacting effects of landscape fragmentation, hunting pressure, translocations, contact with domestic pigs, and disease-related demographic changes. Although STR data can reveal management-relevant differentiation and host connectivity, conclusions regarding deep evolutionary history, complex introgression, or disease transmission require complementary genomic, ecological, and epidemiological evidence. This positions microsatellites as a practical monitoring layer within integrative management rather than as a stand-alone solution.

## References

[cit0001] Alexandri P, Megens HJ, Crooijmans RPMA, Groenen MAM, Goedbloed DJ, Herrero-Medrano JM, Rund LA, Schook LB, Chatzinikos E, Triantaphyllidis C, et al. 2017. Distinguishing migration events of different timing for wild boar in the Balkans. J Biogeogr. 44: 259–270. 10.1111/jbi.12861.

[cit0002] Alkhamis MA, Gallardo C, Jurado C, Soler A, Arias M, Sánchez-Vizcaíno JM. 2018. Phylodynamics and evolutionary epidemiology of African swine fever p72-CVR genes in Eurasia and Africa. PLoS One 13: e0192565. 10.1371/JOURNAL.PONE.0192565. PMC5831051 29489860

[cit0003] Anderson D, Negishi Y, Ishiniwa H, Okuda K, Hinton TG, Toma R, Nagata J, Tamate HB, Kaneko S. 2021. Introgression dynamics from invasive pigs into wild boar following the March 2011 natural and anthropogenic disasters at Fukushima. Proc R Soc B. 288: 20210874. 10.1098/rspb.2021.0874. PMC8242833 34187197

[cit0004] Anderson D, Negishi Y, Toma R, Nagata J, Tamate H, Kaneko S. 2020. Robust microsatellite markers for hybrid analysis between domesticated pigs and wild boar: markers for pig and wild boar hybridization. Genet Resour. 1: 29–41. 10.46265/genresj.BNHB8715.

[cit0005] Anderson EC, Thompson EA. 2002. A model-based method for identifying species hybrids using multilocus genetic data. Genetics. 160: 1217–1229. 10.1093/GENETICS/160.3.1217. PMC1462008 11901135

[cit0006] Antão-Sousa S, Pinto N, Rende P, Amorim A, Gusmão L. 2023. The sequence of the repetitive motif influences the frequency of multistep mutations in short tandem repeats. Sci Rep. 13: 10251. 10.1038/s41598-023-32137-y. PMC10290632 37355683

[cit0007] Bagshaw ATM. 2017. Functional mechanisms of microsatellite DNA in eukaryotic genomes. Genome Biol Evol. 9: 2428–2443. 10.1093/gbe/evx164. PMC5622345 28957459

[cit0008] Blouin MS. 2003. DNA-based methods for pedigree reconstruction and kinship analysis in natural populations. Trends Ecol Evol. 18: 503–511. 10.1016/S0169-5347(03)00225-8.

[cit0009] Böheim D, Knauer F, Stefanović M, Zink R, Kübber-Heiss A, Posautz A, Beiglböck C, Dressler A, Strauss V, Dier H, et al. 2023. Signals of pig ancestry in wild boar, *Sus scrofa*, from eastern Austria: current hybridisation or incomplete gene pool differentiation and historical introgressions? Diversity 15: 790. 10.3390/d15060790.

[cit0010] Bonin A, Bellemain E, Eidesen PB, Pompanon F, Brochmann C, Taberlet P. 2004. How to track and assess genotyping errors in population genetics studies. Mol Ecol. 13: 3261–3273. 10.1111/J.1365-294X.2004.02346.X. 15487987

[cit0011] Bruford MW, Wayne RK. 1993. Microsatellites and their application to population genetic studies. Curr Opin Genet Dev. 3: 939–943. 10.1016/0959-437X(93)90017-J. 8118220

[cit0012] Chapuis MP, Estoup A. 2007. Microsatellite null alleles and estimation of population differentiation. Mol Biol Evol. 24: 621–631. 10.1093/MOLBEV/MSL191. 17150975

[cit0013] Choi SK, Lee JE, Kim YJ, Min MS, Voloshina I, Myslenkov A, Oh JG, Kim TH, Markov N, Seryodkin I, et al. 2014. Gene-tic structure of wild boar (*Sus scrofa*) populations from East Asia based on microsatellite loci analyses. BMC Genet. 15: 85. 10.1186/1471-2156-15-85. PMC4112609 25034725

[cit0014] Cornuet JM, Luikart G. 1996. Description and power analysis of two tests for detecting recent population bottlenecks from allele frequency data. Genetics 144: 2001. 10.1093/genetics/144.4.2001. PMC1207747 8978083

[cit0015] Costa V, Pérez-González J, Santos P, Fernández-Llario P, Carranza J, Zsolnai A, Anton I, Buzgá J, Varga G, Monteiro N, et al. 2012. Microsatellite markers for identification and parentage analysis in the European wild boar (*Sus scrofa*). BMC Res Notes 5: 479. 10.1186/1756-0500-5-479. PMC3475110 22943565

[cit0016] Dakin EE, Avise JC. 2004. Microsatellite null alleles in parentage analysis. Heredity. 93: 504–509. 10.1038/SJ.HDY.6800545. 15292911

[cit0017] De Barba M, Miquel C, Lobréaux S, Quenette PY, Swenson JE, Taberlet P. 2017. High-throughput microsatellite genotyping in ecology: improved accuracy, efficiency, standardization and success with low-quantity and degraded DNA. Mol Ecol Resour. 17: 492–507. 10.1111/1755-0998.12594. 27505280

[cit0018] De Jong JF, Iacolina L, Prins HHT, van Hooft P, Crooijmans RPMA, van Wieren SE, Baños JV, Baubet E, Cahill S, Ferreira E, et al. 2023. Spatial genetic structure of European wild boar, with inferences on late-Pleistocene and Holocene demographic history. Heredity 130: 135–144. 10.1038/s41437-022-00587-1. PMC9981775 36639700

[cit0019] Do C, Waples RS, Peel D, Macbeth GM, Tillett BJ, Ovenden JR. 2014. NeEstimator v2: re-implementation of software for the estimation of contemporary effective population size (Ne) from genetic data. Mol Ecol Resour. 14: 209–214. 10.1111/1755-0998.12157. 23992227

[cit0020] Ellegren H. 2004. Microsatellites: simple sequences with complex evolution. Nat Rev Genet. 5: 435–445. 10.1038/NRG1348. 15153996

[cit0021] Estoup A, Jarne P, Cornuet JM. 2002. Homoplasy and mutation model at microsatellite loci and their consequences for population genetics analysis. Mol Ecol. 11: 1591–1604. 10.1046/J.1365-294X.2002.01576.X. 12207711

[cit0022] Evanno G, Regnaut S, Goudet J. 2005. Detecting the number of clusters of individuals using the software STRUCTURE: a simulation study. Mol Ecol. 14: 2611–2620. 10.1111/J.1365-294X.2005.02553.X. 15969739

[cit0023] Fan H, Chu JY. 2007. A brief review of short tandem repeat mutation. Genom Proteom Bioinform. 5: 7. 10.1016/S1672-0229(07)60009-6. PMC5054066 17572359

[cit0024] Frantz AC, Cellina S, Krier A, Schley L, Burke T. 2009. Using spatial Bayesian methods to determine the genetic structure of a continuously distributed population: clusters or isolation by distance? J Appl Ecol. 46: 493–505. 10.1111/J.1365-2664.2008.01606.X.

[cit0025] Frantz AC, Zachos FE, Kirschning J, Cellina S, Bertouille S, Mamuris Z, Koutsogiannouli EA, Burke T. 2013. Genetic evidence for introgression between domestic pigs and wild boars (*Sus scrofa*) in Belgium and Luxembourg: a comparative approach with multiple marker systems. Biol J Linn Soc. 110: 104–115. 10.1111/bij.12111.

[cit0026] Gamelon M, Coudrin JP, Capron G, Gilot A, Baubet É, Cachelou J, Gaillard JM. 2025. Natal dispersal patterns in a social wild mammal: what does family tell us? Ecology 106: e70190. 10.1002/ECY.70190. 40915971

[cit0027] Garza JC, Williamson EG. 2001. Detection of reduction in population size using data from microsatellite loci. Mol Ecol. 10: 305–318. 10.1046/j.1365-294x.2001.01190.x. 11298947

[cit0028] Goedbloed DJ, van Hooft P, Megens HJ, Langenbeck K, Lutz W, Crooijmans RPMA, van Wieren SE, Ydenberg RC, Prins HHT. 2013. Reintroductions and genetic introgression from domestic pigs have shaped the genetic population structure of Northwest European wild boar. BMC Genet. 14: 43. 10.1186/1471-2156-14-43. PMC3663677 23688182

[cit0029] Goicolea T, Cisneros-Araújo P, Vega CA, Sánchez-Vizcaíno JM, Mateo-Sánchez MC, Bosch J. 2024. Landscape connectivity for predicting the spread of ASF in the European wild boar population. Sci Rep. 14: 3414. 10.1038/s41598-024-53869-5. PMC10858927 38341478

[cit0030] Griciuvienė L, Janeliūnas Ž, Jurgelevičius V, Paulauskas A. 2021. The effects of habitat fragmentation on the genetic structure of wild boar (*Sus scrofa*) population in Lithuania. BMC Genom Data 22: 53. 10.1186/s12863-021-01008-8. PMC8626901 34837959

[cit0031] Griciuvienė L, Janeliūnas Ž, Pilevičienė S, Jurgelevičius V, Paulauskas A. 2022. Changes in the genetic structure of Lithuania’s wild boar (*Sus scrofa*) population following the outbreak of African swine fever. Genes 13: 1561. 10.3390/genes13091561. PMC9498859 36140730

[cit0032] Guichoux E, Lagache L, Wagner S, Chaumeil P, Léger P, Lepais O, Lepoittevin C, Malausa T, Revardel E, Salin F, et al. 2011. Current trends in microsatellite genotyping. Mol Ecol Resour. 11: 591–611. 10.1111/j.1755-0998.2011.03014.x. 21565126

[cit0033] Hale ML, Burg TM, Steeves TE. 2012. Sampling for microsatellite-based population genetic studies: 25 to 30 individuals per population is enough to accurately estimate allele frequencies. PLoS One 7: e45170. 10.1371/JOURNAL.PONE. PMC3440332 22984627

[cit0034] Hauser SS, Athrey G, Leberg PL. 2021. Waste not, want not: microsatellites remain an economical and informative technology for conservation genetics. Ecol Evol. 11: 15800. 10.1002/ece3.8250. PMC8601879 34824791

[cit0035] Holderegger R, Wagner HH. 2008. Landscape genetics. Bio-Science 58: 199–207. 10.1641/B580306.

[cit0036] Johansson AM, Dalin AM, Jonas E, Mikko S, Malmsten A. 2026. Wild or tame? In search for the genetic origin of wild boar (*Sus scrofa*) in Sweden. Ecol Evol. 16: e73369. 10.1002/ece3.73369. PMC13052102 41948220

[cit0037] Jombart T, Devillard S, Balloux F. 2010. Discriminant analysis of principal components: a new method for the analysis of genetically structured populations. BMC Genet. 11: 94. 10.1186/1471-2156-11-94. PMC2973851 20950446

[cit0038] Jones OR, Wang J. 2010. COLONY: a program for parentage and sibship inference from multilocus genotype data. Mol Ecol Resour. 10: 551–555. 10.1111/J.1755-0998.2009.02787.X. 21565056

[cit0039] Kierepka EM, Unger SD, Keiter DA, Beasley JC, Rhodes OE, Cunningham FL, Piaggio AJ. 2016. Identification of robust microsatellite markers for wild pig fecal DNA. J Wildl Manag. 80: 1120–1128. 10.1002/jwmg.21102.

[cit0040] Kruglyak S, Durrett RT, Schug MD, Aquadro CF. 1998. Equilibrium distributions of microsatellite repeat length resulting from a balance between slippage events and point mutations. Proc Natl Acad Sci USA 95: 10774–10778. 10.1073/pnas.95.18.10774. PMC27971 9724780

[cit0041] Kusza S, Podgórski T, Scandura M, Borowik T, Jávor A, Sidorovich VE, Bunevich AN, Kolesnikov M, Jêdrzejewska B. 2014. Contemporary genetic structure, phylogeography and past demographic processes of wild boar *Sus scrofa* population in Central and Eastern Europe. PLoS One 9: e91401. 10.1371/journal.pone.0091401. PMC3951376 24622149

[cit0042] Lecis R, Dondina O, Orioli V, Biosa D, Canu A, Fabbri G, Iacolina L, Cossu A, Bani L, Apollonio M, et al. 2022. Main roads and land cover shaped the genetic structure of a Mediterranean island wild boar population. Ecol Evol. 12: e8804. 10.1002/ece3.8804. PMC8986547 35414901

[cit0043] Lepais O, Chancerel E, Boury C, Salin F, Manicki A, Taillebois L, Dutech C, Aissi A, Bacles CFE, Daverat F, et al. 2020. Fast sequence-based microsatellite genotyping development workflow. PeerJ. 2020: e9085. 10.7717/peerj.9085. PMC7204839 32411534

[cit0044] Levinson G, Gutman GA. 1987. Slipped-strand mispairing: a major mechanism for DNA sequence evolution. Mol Biol Evol. 4: 203–221. 10.1093/oxfordjournals.molbev.a040442. 3328815

[cit0045] Manel S, Gaggiotti OE, Waples RS. 2005. Assignment methods: matching biological questions with appropriate techniques. Trends Ecol Evol. 20: 136–142. 10.1016/j.tree.2004.12.004. 16701357

[cit0046] Manel S, Schwartz MK, Luikart G, Taberlet P. 2003. Landscape genetics: combining landscape ecology and population genetics. Trends Ecol Evol. 18: 189–197. 10.1016/S0169-5347(03)00008-9.

[cit0047] Marshall TC, Slate J, Kruuk LEB, Pemberton JM. 1998. Statistical confidence for likelihood-based paternity inference in natural populations. Mol Ecol. 7: 639–655. 10.1046/J.1365-294X.1998.00374.X. 9633105

[cit0048] Mary N, Iannuccelli N, Petit G, Bonnet N, Pinton A, Barasc H, Faure A, Calgaro A, Grosbois V, Servin B, et al. 2022. Genome-wide analysis of hybridization in wild boar populations reveals adaptive introgression from domestic pig. Evol Appl. 15: 1115–1128. 10.1111/eva. PMC9309462 35899256

[cit0049] Massei G, Kindberg J, Licoppe A, Gačić D, Šprem N, Kamler J, Baubet E, Hohmann U, Monaco A, Ozoliņš J, et al. 2015. Wild boar populations up, numbers of hunters down? A review of trends and implications for Europe. Pest Manag Sci. 71: 492–500. 10.1002/PS.3965. 25512181

[cit0050] Mazloum A, van Schalkwyk A, Shotin A, Zinyakov N, Igolkin A, Chernishev R, Debeljak Z, Korennoy F, Sprygin AV. 2023. Whole-genome sequencing of African swine fever virus from wild boars in the Kaliningrad region reveals unique and distinguishing genomic mutations. Front Vet Sci. 9: 1019808. 10.3389/FVETS.2022.1019808/TEXT. PMC9849583 36686186

[cit0051] Meirmans PG. 2012. The trouble with isolation by distance. Mol Ecol. 21: 2839–2846. 10.1111/J.1365-294X.2012.05578.X. 22574758

[cit0052] Meirmans PG. 2015. Seven common mistakes in population genetics and how to avoid them. Mol Ecol. 24: 3223–3231. 10.1111/MEC.13243. 25974103

[cit0053] Meletiadis A, Garcia-Vozmediano A, Riina MV, Vitale N, Di Nicola MR, Maurella C, Massimino M, Caniglia R, Moroni B, Viani A, et al. 2025. Genetic connectivity and admixture zones shape the spread of African swine fever in wild boar populations in North-western Italy. Sci Rep. 16: 2731. 10.1038/s41598-025-32491-z. PMC12824347 41408392

[cit0054] Mihalik B, Frank K, Astuti PK, Szemethy D, Szendrei L, Szemethy L, Kusza S, Stéger V. 2020. Population genetic structure of the wild boar (*Sus scrofa*) in the Carpathian Basin. Genes 11: 1194. 10.3390/genes11101194. PMC7602151 33066463

[cit0055] Niedziałkowska M, Tarnowska E, Ligmanowska J, Jêdrzejewska B, Podgórski T, Radziszewska A, Ratajczyk I, Kusza S, Bunevich AN, Danila G, et al. 2021. Clear phylogeographic pattern and genetic structure of wild boar *Sus scrofa* population in Central and Eastern Europe. Sci Rep. 11. 10.1038/s41598-021-88991-1. PMC8102581 33958636

[cit0056] Osborne MJ, Caeiro-Dias G, Turner TF. 2023. Transitioning from microsatellites to SNP-based microhaplotypes in genetic monitoring programmes: lessons from paired data spanning 20 years. Mol Ecol. 32: 316–334. 10.1111/mec.16760. 36321869

[cit0057] Paetkau D, Slade R, Burden M, Estoup A. 2004. Genetic assignment methods for the direct, real-time estimation of migration rate: a simulation-based exploration of accuracy and power. Mol Ecol. 13: 55–65. 10.1046/J.1365-294X.2004.02008.X. 14653788

[cit0058] Peery ZM, Kirby R, Reid BN, Stoelting R, Doucet-Bëer E, Robinson S, Vásquez-Carrillo C, Pauli JN, Palsboll PJ. 2012. Reliability of genetic bottleneck tests for detecting recent population declines. Mol Ecol. 21: 3403–3418. 10.1111/j.1365-294X.2012.05635.x. 22646281

[cit0059] Pepin KM, Golnar A, Podgórski T. 2021. Social structure defines spatial transmission of African swine fever in wild boar. J R Soc Interface 18: 20200761. 10.1098/rsif.2020.0761. PMC7879769 33468025

[cit0060] Podgórski T, Lusseau D, Scandura M, Sönnichsen L, Jêdrzejewska B. 2014. Long-lasting, kin-directed female interactions in a spatially structured wild boar social network. PLoS One 9: e99875. 10.1371/journal.pone.0099875. PMC4053407 24919178

[cit0061] Poteaux C, Baubet E, Kaminski G, Brandt S, Dobson FS, Baudoin C. 2009. Socio-genetic structure and mating system of a wild boar population. J Zool. 278: 116–125. 10.1111/j.1469-7998.2009.00553.x.

[cit0062] Pritchard JK, Stephens M, Donnelly P. 2000. Inference of population structure using multilocus genotype data. Genetics 155: 945–959. 10.1093/GENETICS/155.2.945. PMC1461096 10835412

[cit0063] Puechmaille SJ. 2016. The program structure does not reliably recover the correct population structure when sampling is uneven: subsampling and new estimators alleviate the problem. Mol Ecol Resour. 16: 608–627. 10.1111/1755-0998.12512. 26856252

[cit0064] Putman AI, Carbone I. 2014. Challenges in analysis and interpretation of microsatellite data for population genetic studies. Ecol Evol. 4: 4399. 10.1002/ece3.1305. PMC4267876 25540699

[cit0065] Rannala B, Mountain JL. 1997. Detecting immigration by using multilocus genotypes. Proc Natl Acad Sci USA 94: 9197–9201. 10.1073/pnas.94.17.9197. PMC23111 9256459

[cit0066] Reinar WB, Krabberød AK, Lalun VO, Butenko MA, Jakobsen KS. 2024. Short tandem repeats delineate gene bodies across eukaryotes. Nat Commun. 15: 10902. 10.1038/s41467-024-55276-w. PMC11686069 39738068

[cit0067] Rodrigáñez J, Barragán C, Alves E, Gortázar C, Toro MA, Silió L. 2008. Genetic diversity and allelic richness in Spanish wild and domestic pig population estimated from microsatellite markers. Span J Agric Res. 6: 107–115. 10.5424/sjar/200806S1-379.

[cit0068] Salih D, Armstrong EE, Robbins CT, Waits LP, Kelley JL. 2025. Bridging the gap between legacy PCR-based microsatellite data with high-throughput sequencing data in conservation genomics. J Hered. 10.1093/jhered/esaf090. PMC13539450 41182241

[cit0069] Šarhanová P, Pfanzelt S, Brandt R, Himmelbach A, Blattner FR. 2018. SSR-seq: genotyping of microsatellites using next-generation sequencing reveals higher level of polymorphism as compared to traditional fragment size scoring. Ecol Evol. 8: 10817. 10.1002/ECE3.4533. PMC6262739 30519409

[cit0070] Sawai K, Arakawa A, Taniguchi M, Xiao B, Sawai M, Osaki M, Yamaguchi E, Hayama Y, Murato Y, Shimizu Y, et al. 2023. Assessing population structure and migration patterns of wild boar (*Sus scrofa*) in Japan. Sci Rep. 13: 21186. 10.1038/s41598-023-48215-0. PMC10692317 38040788

[cit0071] Scandura M, Iacolina L, Apollonio M. 2011. Genetic diversity in the European wild boar *Sus scrofa*: phylogeography, population structure and wild × domestic hybridization. Mamm Rev. 41: 125–137. 10.1111/j.1365-2907.2010.00182.x.

[cit0072] Scandura M, Iacolina L, Crestanello B, Pecchioli E, Di Benedetto MF, Russo V, Davoli R, Apollonio M, Bertorelle G. 2008. Ancient vs. recent processes as factors shaping the genetic variation of the European wild boar: are the effects of the last glaciation still detectable? Mol Ecol. 17: 1745–1762. 10.1111/j.1365-294X.2008.03703.x. 18371016

[cit0073] Schleimer A, Richart L, Drygala F, Casabianca F, Maestrini O, Weigand H, Schwartz C, Mittelbronn M, Frantz AC. 2022. Introgressive hybridisation between domestic pigs (*Sus scrofa domesticus*) and endemic Corsican wild boars (*S. s. meridionalis*): effects of human-mediated interventions. Heredity 128: 279–290. 10.1038/s41437-022-00517-1. PMC8986821 35273382

[cit0074] Schlotterer C. 2000. Evolutionary dynamics of microsatellite DNA. Chromosoma 109: 365–371. 10.1007/s004120000089. 11072791

[cit0075] Selkoe KA, Toonen RJ. 2006. Microsatellites for ecologists: a practical guide to using and evaluating microsatellite markers. Ecol Lett. 9: 615–629. 10.1111/J.1461-0248.2006.00889.X. 16643306

[cit0076] Simon U, Gerhards K, Becker S, Willems H, Friedrichs V, Forth JH, Calvelage S, Blome S, Reiner G. 2024. Genetic differentiation of wild boar populations in a region affected by African swine fever. Eur J Wildl Res. 70: 54. 10.1007/s10344-024-01807-1.

[cit0077] Storfer A, Murphy MA, Evans JS, Goldberg CS, Robinson S, Spear SF, Dezzani R, Delmelle E, Vierling L, Waits LP. 2006. Putting the ‘landscape’ in landscape genetics. Heredity 98: 128–142. 10.1038/sj.hdy.6800917. 17080024

[cit0078] Vähä JP, Primmer CR. 2006. Efficiency of model-based Bayesian methods for detecting hybrid individuals under different hybridization scenarios and with different numbers of loci. Mol Ecol. 15: 63–72. 10.1111/J.1365-294X.2005.02773.X. 16367830

[cit0079] Van Oosterhout C, Hutchinson WF, Wills DPM, Shipley P. 2004. Micro-checker: software for identifying and correcting genotyping errors in microsatellite data. Mol Ecol Notes 4: 535–538. 10.1111/j.1471-8286.2004.00684.x.

[cit0080] Veličković N, Ferreira E, Djan M, Ernst M, Obreht Vidaković D, Monaco A, Fonseca C. 2016. Demographic history, current expansion and future management challenges of wild boar populations in the Balkans and Europe. Heredity 117: 348–357. 10.1038/hdy.2016.53. PMC5061920 27436523

[cit0081] Waits LP, Paetkau D. 2005. Noninvasive genetic sampling tools for wildlife biologists: a review of applications and recommendations for accurate data collection. J Wildl Manag. 69: 1419–1433. 10.2193/0022-541X(2005)69[1419:NGSTFW]2.0.CO;2.

[cit0082] Wang J. 2011. Coancestry: a program for simulating, estimating and analysing relatedness and inbreeding coefficients. Mol Ecol Resour. 11: 141–145. 10.1111/j.1755-0998.2010.02885.x. 21429111

[cit0083] Wang Z, Li Z, Huang T, Chen J, Xu P, Qiao R, Yin H, Song C, Zhang D, Liu D, et al. 2025. Genomic insights into the demographic history and local adaptation of wild boars across Eurasia. Cell Genom. 5: 100954. 10.1016/j.xgen.2025.100954. PMC12534701 40712570

[cit0084] Waples RS, Do C. 2010. Linkage disequilibrium estimates of contemporary Ne using highly variable genetic markers: a largely untapped resource for applied conservation and evolution. Evol Appl. 3: 244–262. 10.1111/J.1752-4571.2009.00104.X. PMC3352464 25567922

[cit0085] Wright SE, Todd PK. 2023. Native functions of short tandem repeats. eLife 12: e84043. 10.7554/eLife.84043. PMC10027321 36940239

[cit0086] Zemanova MA. 2021. Noninvasive genetic assessment is an effective wildlife research tool when compared with other approaches. Genes 12: 1672. 10.3390/genes12111672. PMC8625682 34828277

